# Multi-Omics Analysis Showed the Clinical Value of Gene Signatures of C1QC^+^ and SPP1^+^ TAMs in Cervical Cancer

**DOI:** 10.3389/fimmu.2021.694801

**Published:** 2021-07-06

**Authors:** Xiong Li, Qinghua Zhang, Gang Chen, Danfeng Luo

**Affiliations:** ^1^ Department of Obstetrics and Gynecology, Tongji Hospital, Tongji Medical College, Huazhong University of Science and Technology, Wuhan, China; ^2^ Department of Obstetrics and Gynecology, the Central Hospital of Wuhan, Tongji Medical College, Huazhong University of Science and Technology, Wuhan, China

**Keywords:** cervical cancer, TAMs (tumor associated myeloid cells), C1QC, SPP1 gene, single cell, immunity

## Abstract

**Purpose:**

To evaluate the value of C1QC^+^ and SPP1^+^ TAMs gene signatures in patients with cervical cancer.

**Methods:**

We compare the C1QC^+^ and SPP1^+^ TAMs gene signatures with the M1/M2 gene signatures at single cell level and bulk RNA-seq level and evaluate which gene signature can clearly divide TAMs and patients with cervical cancer into distinct clinical subclusters better.

**Results:**

At single-cell level, C1QC^+^ and SPP1^+^ TAMs gene signatures, but not M1 and M2 gene signatures, could clearly divided TAMs into two subclusters in a colon cancer data set and an advanced basal cell data set. For cervical cancer data from TCGA, patients with C1QC^high^ and SPP1^low^ TAMs gene signatures have the best prognosis, lowest proportion (34.21%) of locally advanced cervical cancer (LACC), and highest immune cell infiltration, whereas patients with C1QC^low^ and SPP1^high^ TAMs gene signatures have the worst prognosis, highest proportion (71.79%) of LACC and lowest immune cell infiltration. Patients with C1QC^high^ and SPP1^low^ TAMs gene signature have higher expression of most of the Immune checkpoint molecules (ICMs) than patients with C1QC^low^ and SPP1^high^ TAMs gene signatures. The GSEA results suggested that subgroups of patients divided by C1QC^+^ and SPP1^+^ TAMs gene signatures showed different anti- or pro-tumor state.

**Conclusion:**

C1QC^+^ and SPP1^+^ TAMs gene signatures, but not M1/M2 gene signatures, can divide cervical patients into subgroups with different prognosis, tumor stage, different immune cell infiltration, and ICMs expression. Our findings may help to find suitable treatment strategy for cervical cancer patients with different TAMs gene signatures.

## Introduction

Despite initiatives to improve the prevention of cervical cancer with screening and vaccination, cervical cancer is still one of the leading causes of death among women worldwide (1). Improvements in survival have mainly been through effective surgery, technical radiotherapy, and addition of bevacizumab to standard chemotherapy in recent years (2, 3). However, women with advanced or recurrent disease still face a dismal prognosis with potentially considerable morbidity and mortality. Immunotherapy might be a novel choice to improve the clinical outcomes of these patients. On the established clinical benefit of PD-1/PD-L1 inhibitors in cervical cancer, the Food and Drug Administration (FDA) has approved pembrolizumab for patients with recurrent or metastatic cervical cancer with disease progression during or after chemotherapy. However, treatment options are still limited, extensive researches and clinical trials are needed to be carried out to identify novel Immunotherapy signatures and options (4, 5).

The tumor microenvironment (TME) are governed by crosstalks within and across various cellular compartments, including immune, malignant, endothelial, and stromal cells (6). Tumor-associated macrophages (TAMs), which are considered as the main components of the tumor microenvironment, reportedly play key roles in the initiation and progression of cancers (7, 8). The TAMs are highly dynamic and heterogeneous within and across different cancers (6, 9). TAMs’ heterogeneity makes them with various functions. Different subsets of TAMs may show distinct functions. However, the distinction of different subsets of TAMs varied in different studies. In lung cancer and breast cancer, TAMs reportedly showed a continuous spectrum of phenotypes (10–12). In some other cancers, TAMs were classified into “traditional” pro-inflammatory (M1-like) or anti-inflammatory (M2-like) TAMs (6, 13). However, Lei et al. (10) reported that TAMs in colon cancer exhibited a remarkable dichotomy and were defined as C1QC^+^ TAMs and SPP1^+^ TAMs. Besides, the C1QC^+^ TAMs and SPP1^+^ TAMs could not be explained by the expression analyses based on genes associated with M1 and M2 TAMs in the colon cancer. The tumor angiogenesis, cell migration, ECM receptor interaction, and tumor vasculature pathways were enriched in SPP1^+^ TAMs, whereas the complement activation and antigen processing and presentation pathways were significantly enriched in C1QC^+^ TAMs. In addition, the combination of C1QC^+^ and SPP1^+^ TAMs gene signatures could separate patients from TCGA COAD and READ into subgroups of distinct prognosis. Based on that, patients with C1QC^high^ and SPP1^low^ TAMs gene signatures had the best prognosis, whereas patients with C1QC^low^ and SPP1^high^ TAMs gene signatures had the worst prognosis.

In different stages of cervical cancer, the phenotype of macrophages is constantly changing, which affects the ability of proliferation, invasion, and metastasis of cancer cells in many ways (14, 15). The number of TAMs in cervical lesion matrix changes with the progress of cervical cancer. However, whether TAMs in cervical cancer show as the M1 and M2 phenotypes or C1QC^+^ and SPP1^+^ TAMs phenotypes remains unknown. It is best to use the single cell sequencing technology to distinct subsets of TAMs of cervical cancer; however, there is no single cell sequencing database in cervical cancer to be used so far. However, we can use bulk transcriptome data of cervical patients from TCGA to evaluate the gene signatures of known TAMs subsets.

In this study, we compared the C1QC^+^ and SPP1^+^ TAMs gene signatures, as well as classic M1 and M2 gene signatures, using transcriptome data of TCGA cervical cancer patients. We aim to find the relationship between different TAMs gene signatures and clinical features and the mechanisms behind, which may provide suggestion to treatment of cervical cancer in clinic.

## Materials and Methods

### Sources for Single Cell Data, Bulk RNA-Seq Data, and Immune Cell Infiltration Estimation of TCGA Samples

Processed single-cell data of colon cancer was obtained from Gene Expression Omnibus (GEO) (GSE146771) (10). While processed single-cell data of advanced basal cell carcinoma was obtained from GEO (GSE123814) (16).

Bulk RNA-seq gene expression data and clinical data of cervical cancer were downloaded from UCSC Xena (https://xenabrowser.net/datapages/). The bulk RNA-seq gene expressions were log2(TPM+1) transformed. Immune cell infiltration estimation of TCGA samples were downloaded from TIMER2.0 (http://timer.cistrome.org/), which included immune signatures of TCGA samples calculated using TIMER, CIBERSORT, and xCell (17). Tumor mutational burden (TMB) data of TCGA samples were obtained from Vésteinn et al.’s study (18).

### Define C1QC^+^ TAMs, SPP1^+^ TAMs, and M1/M2 Gene Signatures

C1QC^+^ TAMs and SPP1^+^ TAMs gene signatures defined in Zhang et al.’s study were used in our paper (10). C1QC^+^ TAMs gene signature include the following genes: C1QA, C1QB, ITM2B, C1QC, HLA-DMB, MS4A6A, CTSC, TBXAS1, TMEM176B, SYNGR2, ARHGDIB, TMEM176A, UCP2, CAPZB, MAF, TREM2, and MSR1, whereas SPP1^+^ TAMs gene signature includes the following genes: SPP1, PCSK5, SLC11A1, VCAN, SLC25A37, FLNA, UPP1, BCL6, AQP9, TIMP1, VEGFA, ADM, MARCO, FN1, and IL1RN.

The M1/M2 gene signatures were obtained from Azizi et al.’s research (10, 11). Genes associated with “classically activated” (M1) macrophages include CCL5, CCR7, CD40, CD86, CXCL9, CXCL10, CXCL11, IDO1, IL1A, IL1B, IL6, IRF1, IRF5, and KYNU, while CCL4, CCL13, CCL18, CCL20, CCL22, CD276, CLEC7A, CTSA, CTSB, CTSC, CTSD, FN1, IL4R, IRF4, LYVE1, MMP9, MMP14, MMP19, MSR1, TGFB1, TGFB2, TGFB3, TNFSF8, TNFSF12, VEGFA, VEGFB, and VEGFC were used to define the signature of “alternatively activated” (M2) macrophages (10).

### Single-Cell Data Analysis

Processed single-cell RNA-seq data were obtained as described above. The annotation information of cell types were included in the metadata as described by the original articles (10, 16). The Seurat v3 (version 3.2.2) R package was used to analyze the processed scRNA-seq data (19). The function AddModuleScore in Seurat was used to calculate C1QC^+^ TAMs, SPP1^+^ TAMs, and M1/M2 gene signatures using their gene sets, respectively.

### TCGA Bulk RNA-Seq Data Analysis

For the bulk RNA-seq data of TCGA cervical cancer samples, the mean expression of genes in the given signatures (C1QC^+^ TAMs, SPP1^+^ TAMs, and M1/M2 gene signatures) were used as the signature scores. Also, the mean expression of given signatures was grouped into high and low expression groups by the 55^th^ and 45^th^ quantile values (10). Immune cell infiltration estimation of TCGA samples was visualized as heatmaps using the R package ComplexHeatmap (20). Immunotherapy responses were predicted by TIDE (Tumor Immune Dysfunction and Exclusion) as described in a previous study (21).

### Gene Set Enrichment Analysis

Different gene expression between patients with C1QC^high^ and SPP1^low^ TAMs gene signatures and patients with C1QC^low^ and SPP1^high^ TAMs gene signatures were calculated with LIMMA (version 3.46.0) package. Sorted (by log fold change) different expression gene list was used to perform the gene set enrichment analysis (GSEA) by using clusterProfiler (version 3.18.0) package (22).

### Statistical Analysis

Either Pearson’s chi-square test or Fisher’s exact test was used to assess the different clinicopathological factors according to the different C1QC^+^ TAMs, SPP1^+^ TAMs gene signatures groups. Wilcoxon signed-rank test was used to compare gene and gene signatures between different group of patients. Kaplan-Meier survival curves among different groups were plotted using R function ggsurvplot. Cox proportional hazards model implemented in the R package survival was used to find the predict factors of prognostic. All statistical analyses were performed using R (v4.0.3). All figures were plotted by using R. P values <0.05 were considered as statistically significant difference.

## Results

### C1QC^+^ TAMs and SPP1^+^ TAMs Gene Signatures Can Divide TAMs Into Two Different Subsets in Colon Cancer and Advanced Basal Cell Carcinoma

**Figure 1:**
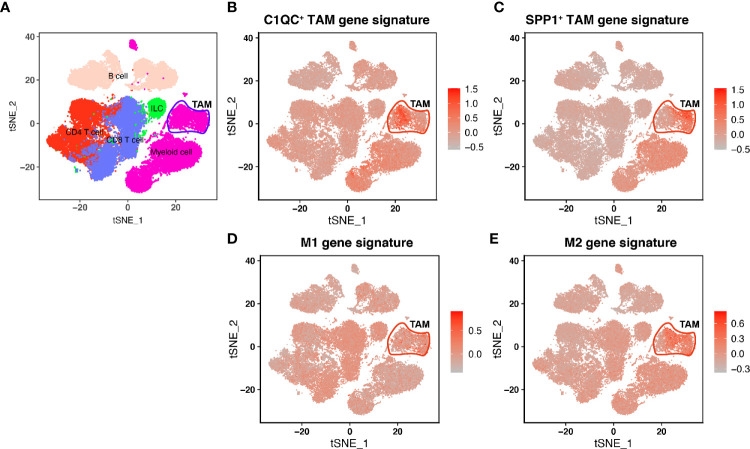
Single-cell transcriptome profiling and TAM gene signatures of the Human CRC TME. **(A)** tSNE plot showing major immune cell subsets in human CRC TME. **(B)** tSNE plot of all immune cells colored by enrichment of C1QC^+^ TAM gene signatures. **(C)** tSNE plot of all immune cells colored by enrichment of SPP1^+^ TAM gene signatures. **(D)** tSNE plot of all immune cells colored by enrichment of M1 gene signatures. **(E)** tSNE plot of all immune cells colored by enrichment of M2 gene signatures.

In Lei’s paper (10), they found that TAMs showed a remarkable dichotomy and could be marked as C1QC^+^ TAMs and SPP1^+^ TAMs. Also, the C1QC^+^ TAMs and SPP1^+^ TAMs were different from “classically activated” M1 and “alternatively activated” M2 macrophages. We used single-cell data from Lei’s paper and found that C1QC^+^ TAMs gene signature and SPP1^+^ TAMs gene signatures have high expressions in two different TAMs subsets, respectively (**Figure 1**), whereas M1 and M2 gene signatures did not have high expressions in different subsets of TAMs (**Figure 1**). To validate if C1QC^+^ TAMs and SPP1^+^ TAMs gene signatures can work better than M1 and M2 signatures in other cancers, we also analyzed another single cell data of advanced basal cell carcinoma (BCC) (16). In the BCC data, C1QC^+^ TAMs and SPP1^+^ TAMs gene signatures, but not M1 and M2 gene signatures, can divide TAMs into two different subsets (**Figure S1**). It is worth mentioning that, in both single cell databases, both C1QC^+^ TAMs and SPP1^+^ TAMs gene signatures had the highest expression only in TAMs but not in other cell types (**Figures 1** and **S1**). These data indicated that at least in colon cancer and advanced basal cell carcinoma, C1QC^+^ TAMs and SPP1^+^ TAMs gene signatures are better separators than M1 and M2 gene signatures to divide TAMs into different subsets, which may represent different immune functions.

### C1QC^+^ TAMs and SPP1^+^ TAMs Gene Signatures Can Divide Cervical Patients Into Different Prognostic and Clinical Subgroups

**Figure 2:**
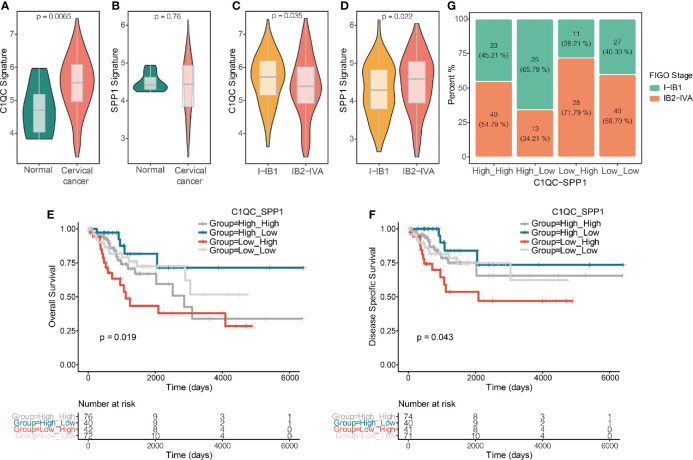
C1QC+ and SPP1+ TAMs gene signatures in TCGA cervical cancer patients. **(A)** Violin plots showing comparison of C1QC^+^ TAM gene signatures levels between normal and cervical cancer samples in TCGA. Two-sided Wilcoxon test. **(B)** Violin plots showing comparison of SPP1^+^ TAM gene signatures levels between normal and cervical cancer samples in TCGA. Two-sided Wilcoxon test. **(C)** Violin plots showing comparison of C1QC^+^ TAM gene signatures levels between patients with FIGO stage 1-IB1 and patients with FIGO stage IB2-IVA in TCGA. Two-sided Wilcoxon test. **(D)** Violin plots showing comparison of SPP1^+^ TAM gene signatures levels between patients with FIGO stage 1-IB1 and patients with FIGO stage IB2-IVA in TCGA. Two-sided Wilcoxon test. **(E)** The Kaplan-Meier overall survival curves of TCGA cervical cancer patients grouped by the gene signature expression of C1QC^+^ TAM and SPP1^+^ TAM. **(F)** The Kaplan-Meier Disease specific survival curves of TCGA cervical cancer patients grouped by the gene signature expression of C1QC^+^ TAM and SPP1^+^ TAM. **(G)** Proportions of patients with FIGO stage I-IB1 and IB2-IVA in cervical cancer patients grouped by the gene signature expression of C1QC^+^ TAM and SPP1^+^ TAM.

Because there is no single-cell database of cervical patients, it is unknown of separation of TAMs from cervical patients into two distinct subgroups based on the TAMs gene signatures. We speculate that if C1QC^+^ TAMs and SPP1^+^ TAMs gene signatures can divide TAMs of cervical cancer patients into two distinct functional subsets, patients with different levels of C1QC^+^ TAMs and SPP1^+^ TAMs gene signatures may have different clinical features. We calculated C1QC^+^ TAMs and SPP1^+^ TAMs gene signatures in cervical cancer patients and normal cervical tissue from TCGA and GTEX, respectively, using their transcriptome data (*Materials and Methods*). Consistent with results in single-cell level data (10), cervical cancer samples showed higher C1QC^+^ TAMs gene signature than normal cervical tissues (**Figure 2A**). However, we did not find significant difference of SPP1^+^ TAMs gene signature between normal cervical tissues and cervical cancer samples (**Figure 2B**). Besides, we found that patients with locally advanced cervical cancer (LACC, Stage IB2-IVA) have lower C1QC^+^ TAMs signature and higher SPP1^+^ TAMs gene signature compared with patients with early stage (stage I-IB1) cervical cancer (**Figures 2C, D** **)**. Although patients with locally advanced cervical cancer and those with early stage cervical cancer have similar M1 and M2 gene signature levels (**Figures S2A, B**).

Next, we divided cervical patients into high and low groups by the 55th and 45th quantile values of C1QC^+^ TAMs and SPP1^+^ TAMs gene signatures, respectively, and further separated patients into four subgroups according to the C1QC^+^ and SPP1^+^ TAMs gene signatures levels. We found patients with C1QC^high^ and SPP1^low^ TAMs gene signatures have the best overall survival (OS) and disease specific survival (DSS) (**Figures 2E, F** **)**, whereas patients with C1QC^low^ and SPP1^high^ TAMs gene signatures have the worst OS and DSS (**Figures 2E, F** **)**. However, M1 and M2 gene signatures could not divide patients into distinct prognosis subgroups (**Figures S2C, D**). We also found that patients with C1QC^high^ and SPP1^low^ TAMs gene signatures have the lowest proportion (34.21%) of LACC, whereas patients with C1QC^low^ and SPP1^high^ TAMs gene signature have the highest proportion (71.79%) of LACC (**Figure 2G**). When comparing clinical features between these two groups, patients with C1QC^low^ and SPP1^high^ TAMs gene signatures had later FIGO stages, more positive pathologic lymph node, higher mortality, and higher proportion of patients developed with disease (**Table 1**). There was no significant difference of histological grade, lymphovascular invasion indicator, tumor status, and metastasis between patients with C1QC^high^ and SPP1^low^ TAMs gene signatures and those with C1QC^low^ and SPP1^high^ TAMs gene signatures (**Table 1**). Besides, after adjusting by age and FIGO stage, the C1QC^low^ and SPP1^high^ TAMs gene signatures showed as an independent predict factor to worse OS (**Table 2**). Although advanced FIGO stage (IB2-IVA) is correlated with C1QC^low^ and SPP1^high^ TAMs gene signatures (**Table 1**), it was not associated with worse prognosis (P = 0.793, **Table 2**). These results suggested that C1QC^+^ and SPP1^+^ TAMs gene signatures could provide additional information besides clinicopathological factors to find cervical patients with different clinical outcome and prognosis.

**Table 1:** Clinicopathological factors of cervical cancer patients from TCGA.

	Overall (N=82)	C1QC^+^-SPP1^+^ TAMs gene signatures		P-value
		High_Low (N=40)	Low_High (N=42)	
**Age, years**				
Mean (SD)	46.0 (13.0)	46.9 (12.9)	45.2 (13.3)	0.567
Median [Min, Max]	44.5 [21.0, 79.0]	44.5 [25.0, 75.0]	44.5 [21.0, 79.0]	
**FIGO Stage**				
I-IB1	36 (43.9%)	25 (62.5%)	11 (26.2%)	0.002
IB2-IVA	41 (50.0%)	13 (32.5%)	28 (66.7%)	
Missing	5 (6.1%)	2 (5.0%)	3 (7.1%)	
**Histological type**				
Adenosquamous	1 (1.2%)	1 (2.5%)	0 (0%)	0.400
Cervical squamous cell carcinoma	69 (84.1%)	31 (77.5%)	38 (90.5%)	
Endocervical adenocarcinoma of the usual type	3 (3.7%)	1 (2.5%)	2 (4.8%)	
Endocervical type of adenocarcinoma	5 (6.1%)	4 (10.0%)	1 (2.4%)	
Endometrioid adenocarcinoma of endocervix	1 (1.2%)	1 (2.5%)	0 (0%)	
Mucinous adenocarcinoma of endocervical type	3 (3.7%)	2 (5.0%)	1 (2.4%)	
**Histological grade**				
G1	5 (6.1%)	2 (5.0%)	3 (7.1%)	0.981
G2	34 (41.5%)	17 (42.5%)	17 (40.5%)	
G3	37 (45.1%)	18 (45.0%)	19 (45.2%)	
GX	6 (7.3%)	3 (7.5%)	3 (7.1%)	
**Lymphovascular invasion indicator**				
Absent	20 (24.4%)	15 (37.5%)	5 (11.9%)	0.126
Present	25 (30.5%)	12 (30.0%)	13 (31.0%)	
Missing	37 (45.1%)	13 (32.5%)	24 (57.1%)	
**Tumor status**				
Tumor free	55 (67.1%)	31 (77.5%)	24 (57.1%)	0.112
With tumor	26 (31.7%)	9 (22.5%)	17 (40.5%)	
Missing	1 (1.2%)	0 (0%)	1 (2.4%)	
**Metastasis**				
No	76 (92.7%)	38 (95.0%)	38 (90.5%)	0.717
Yes	6 (7.3%)	2 (5.0%)	4 (9.5%)	
**Pathologic M**				
M0	30 (36.6%)	19 (47.5%)	11 (26.2%)	0.262
M1	2 (2.4%)	2 (5.0%)	0 (0%)	
MX	34 (41.5%)	17 (42.5%)	17 (40.5%)	
Missing	16 (19.5%)	2 (5.0%)	14 (33.3%)	
**Pathologic N**				
N0	40 (48.8%)	30 (75.0%)	10 (23.8%)	0.002
N1	18 (22.0%)	5 (12.5%)	13 (31.0%)	
NX	10 (12.2%)	4 (10.0%)	6 (14.3%)	
Missing	14 (17.1%)	1 (2.5%)	13 (31.0%)	
**OS**				
No	60 (73.2%)	35 (87.5%)	25 (59.5%)	0.009
Yes	22 (26.8%)	5 (12.5%)	17 (40.5%)	
**DSS**				
No	64 (78.0%)	36 (90.0%)	28 (66.7%)	0.034
Yes	17 (20.7%)	4 (10.0%)	13 (31.0%)	
Missing	1 (1.2%)	0 (0%)	1 (2.4%)	
**DFI**				
No	38 (46.3%)	26 (65.0%)	12 (28.6%)	0.900
Yes	10 (12.2%)	6 (15.0%)	4 (9.5%)	
Missing	34 (41.5%)	8 (20.0%)	26 (61.9%)	
**PFI**				
No	60 (73.2%)	31 (77.5%)	29 (69.0%)	0.539
Yes	22 (26.8%)	9 (22.5%)	13 (31.0%)	
**Treatment**				
Radical surgery	24 (29.3%)	14 (35.0%)	10 (23.8%)	0.771
Radical surgery and radiotherapy, or concurrent chemoradiation	23 (28.0%)	12 (30.0%)	11 (26.2%)	
Radiotherapy	17 (20.7%)	8 (20.0%)	9 (21.4%)	
Other	18 (22.0%)	6 (15.0%)	12 (28.6%)	

TAMs, tumor-associated macrophages; High_Low, C1QC^high^ and SPP1^low^ TAMs gene signatures group; Low_High, C1QC^low^ and SPP1^high^ TAMs gene signatures group; SD, standard deviation; FIGO, International Federation of Gynecology and Obstetrics; OS, overall survival; DSS, disease-specific survival; DFI, disease-free interval; PFI, progression-free interval.

**Table 2:** Prognostic values of clinical factors and C1QC+ and SPP1+ TAMs gene signatures in cervical cancer.

	Overall	HR (univariable)		HR (multivariable)|	
	(n=75)	HR (95% CI)	P	HR (95% CI)	P
**Age, years**					
Mean (SD)	46	1.03 (1.00–1.07)	0.067	1.06 (0.99–1.12)	0.078
**FIGO stage**					
I-IB1	36				
IB2-IVA	41	1.95 (0.79–4.79)	0.146	1.20 (0.30–4.77)	0.793
**Histological type**					
SCC	69				
AS	1	NA	NA	NA	NA
Other	12	0.24 (0.03–1.76)	0.159	0.37 (0.03–4.03)	0.416
**Histological grade**					
G1	5				
G2	34	0.53 (0.07–4.33)	0.554	0.08 (0.00–1.28),	0.074
G3	37	0.90 (0.11–7.21)	0.924	0.03 (0.00–0.91)	0.044
GX	6	4.78 (0.51–45.22)	0.172	0.39 (0.01–12.22)	0.595
**Pathologic M**					
M0	30				
M1	2	2.88 (0.35–23.84)	0.327	NA	NA
MX	34	0.71 (0.26–1.97)	0.514	0.10 (0.01–1.10)	0.059
**Pathologic N**					
N0	40				
N1	18	2.76 (0.84–9.07)	0.094	0.98 (0.20–4.76)	0.981
NX	10	5.48 (1.52–19.76)	0.009	4.91 (0.35–69.22)	0.238
**C1QC_SPP1**					
High_Low	40				
Low_High	42	4.08 (1.50–11.09)	0.006	8.40 (1.33–52.94)	0.023

TAMs, tumor-associated macrophages; HR, hazard ratio; CI, confidence interval; SD, standard deviation; FIGO, International Federation of Gynecology and Obstetrics; SCC, squamous cell carcinoma; AS, adenosquamous cell carcinoma; C1QC_SPP1, C1QC^+^ and SPP1^+^ TAMs gene signatures; High_Low, C1QC^high^ and SPP1^low^ TAMs gene signatures; Low_High, C1QC^low^ and SPP1^high^ TAMs gene signatures.

NA, Not available.

### C1QC^+^ TAMs and SPP1^+^ TAMs Gene Signatures Divide Cervical Patients Into Subgroups With Different Immune States

**Figure 3:**
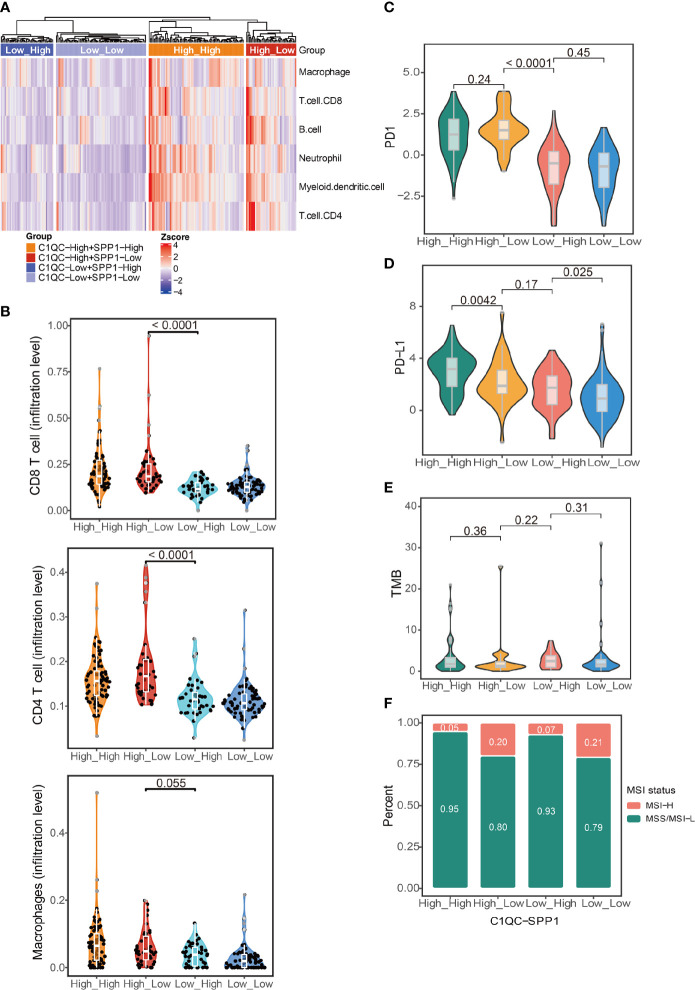
Immune characteristics in different groups of TCGA cervical cancer patients. **(A)** Heatmap showing immune cell signatures by TIMER in cervical cancer patients grouped by the gene signature expression of C1QC^+^ TAM and SPP1^+^ TAM. **(B)** Violin plots showing comparison of CD8 T cell, CD4 T cell, and macrophages gene signatures among cervical cancer patients grouped by the gene signature expression of C1QC^+^ TAM and SPP1^+^ TAM. Two-sided Wilcoxon test. **(C)** Violin plots showing comparison of PD1 gene expression among cervical cancer patients grouped by the gene signature expression of C1QC^+^ TAM and SPP1^+^ TAM. Two-sided Wilcoxon test. **(D)** Violin plots showing comparison of PD-L1 gene expression among cervical cancer patients grouped by the gene signature expression of C1QC^+^ TAM and SPP1^+^ TAM. Two-sided Wilcoxon test. **(E)** Violin plots showing comparison of TMB among cervical cancer patients grouped by the gene signature expression of C1QC^+^ TAM and SPP1^+^ TAM. Two-sided Wilcoxon test. **(F)** Proportions of patients with MSI-H and MSS/MSI-L state in cervical cancer patients grouped by the gene signature expression of C1QC^+^ TAM and SPP1^+^ TAM.

The abundance of different TAM subtypes could have an impact on other immune cells infiltration and disease outcome in patients (6). We compared the immune cell infiltration by using cell type scores calculated by TIMER. Patients with C1QC^high^ and SPP1^low^ TAMs gene signatures had the highest immune cell infiltration, whereas patients with C1QC^low^ and SPP1^high^ TAMs gene signatures had the lowest immune cell infiltration (**Figure 3A**). Also, we found patients with C1QC^high^ and SPP1^low^ TAMs gene signatures had significantly higher CD8 T cell and CD4 T cell infiltration level than patients with C1QC^low^ and SPP1^high^ TAMs gene signatures (**Figure 3B**). The macrophages infiltration level did not show significant difference between patients with C1QC^high^ and SPP1^low^ TAMs gene signatures and patients with C1QC^low^ and SPP1^high^ TAMs gene signatures (**Figure 3B**). This may suggest that it is the different ratio of C1QC^+^ and SPP1^+^ TAMs, but not the TAMs amount, impacts the TME. We also used immune cells infiltration scores calculated by XCELL and CIBERSORT to perform the same analysis, and we found similar results (**Figures S3A, B**). “Hot tumors” which had higher T-cell immune infiltration was reported to have higher response rates to immune checkpoint inhibitors (ICIs) immunotherapies compared with “cold tumors,” which had lower T-cell immune infiltration (23). PD1, PD-L, and tumor mutational burden (TMB) were also reported to be associated with response to ICIs immunotherapy (24). We found that patients with C1QC^high^ TAMs gene signatures had higher PD1 and PD-L1 expression than those with C1QC^low^ TAMs gene signatures (**Figures 3C, D** **)**, and patients with C1QC^high^ and SPP1^low^ TAMs gene signature had the highest PD1 expression compared with the other three subgroups (**Figure 3C**). Also, we found that patients with C1QC^high^ and SPP1^low^ TAMs gene signatures had lowest TMB, whereas patients with C1QC^low^ and SPP1^high^ TAMs gene signatures had highest TMB, although the difference was not significant (**Figure 3E**). Microsatellite instability (MSI) is genetic instability in short nucleotide repeats (microsatellites) because of a high mutation rate resulted in abnormal DNA mismatch repair (25). Tumors with MSI-H exhibit a high mutation rate and neoantigen load that is positively associated with overall lymphocytic infiltration. The tumor-infiltrating lymphocytes, T helper 1 cells and memory T cells, will ultimately trigger an effective antitumor immune response (26–28). MSI only exists in a small subset of cervical cancer patients (29). We found that patients with C1QC^high^ and SPP1^low^ TAMs gene signatures had higher proportion of MSI-H than patients with C1QC^low^ and SPP1^high^ TAMs gene signatures (**Figure 3F**). All these results suggest that patients could be divided into subgroups based on the C1QC^+^ and SPP1^+^ TAMs gene signatures. This distinction is associated with different genomic status, immune cell infiltration, and finally different prognosis, which implies that different ratios of C1QC^+^ and SPP1^+^ TAMs subsets may impact TME state.

### Different Pathways Involved in Different C1QC^+^ and SPP1^+^ TAMs Gene Signatures Subgroups

**Figure 4:**
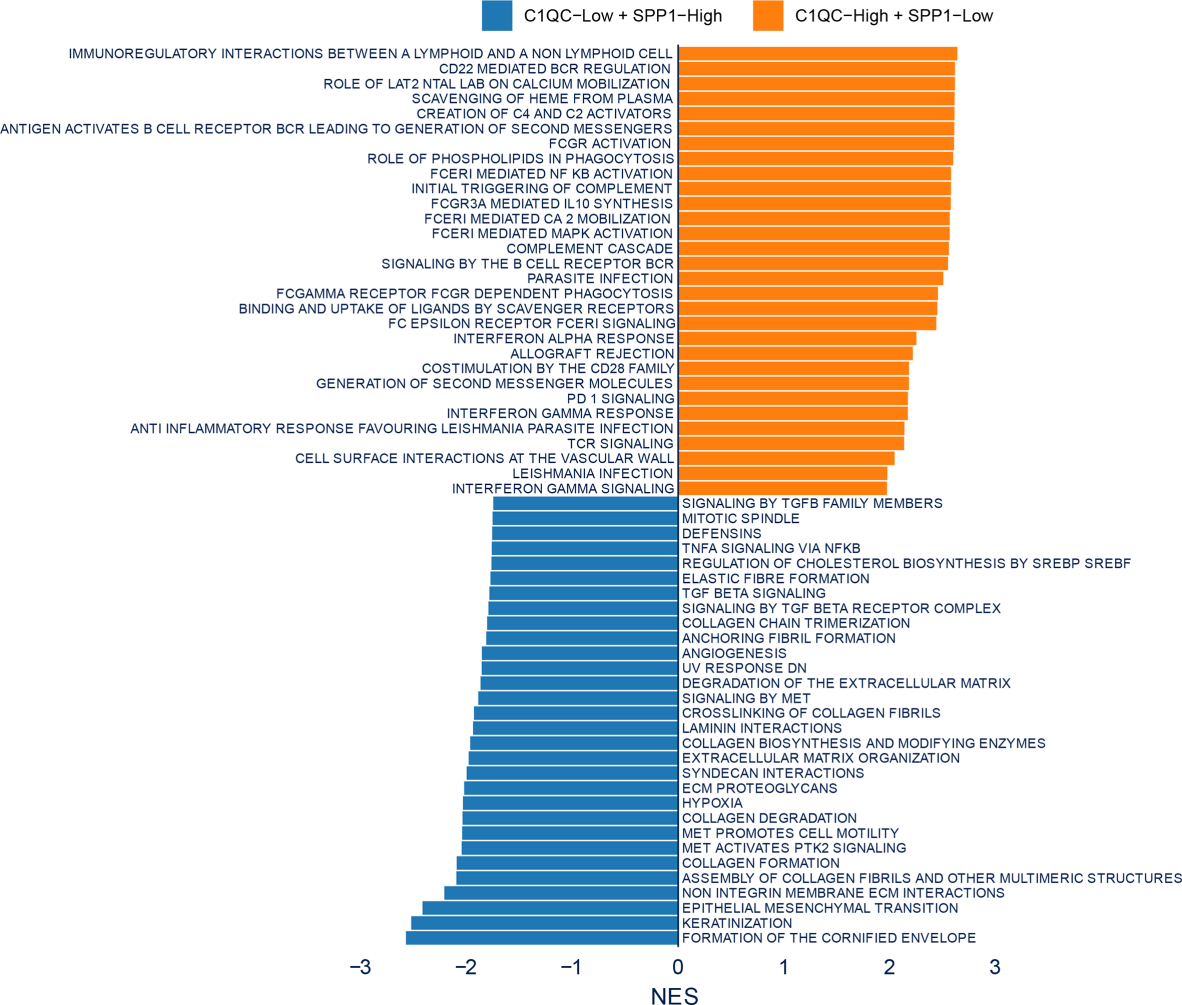
Enrichment plots from gene set enrichment analysis (GSEA). Differential pathway enriched in C1QC^low^ + SPP1^high^ TAMs gene signatures group and C1QC^high^ + SPP1^low^ TAMs gene signatures group.

To figure out if some special pathways involved in different subsets divided by C1QC^+^ and SPP1^+^ TAMs gene signatures, we compared transcriptome data of patients with C1QC^high^ and SPP1^low^ TAMs gene signatures to that of patients with C1QC^low^ and SPP1^high^ TAMs gene signatures. Gene set enrichment analysis (GSEA) was used to detect pathways enriched in different groups. C1QC^high^ and SPP1^low^ TAMs gene signatures group exhibited enrichment of TCR signaling and interferon gamma signaling (**Figure 4**), suggesting the anti-tumor functions in these patients. While C1QC^low^ and SPP1^high^ TAMs gene signatures group exhibited TGFb associated pathways, extracellular matrix organization, and keratinization pathway (**Figure 4**), suggesting the pro-tumorigenic functions in these patients. The GSEA results suggested that subgroups of patients divided by C1QC^+^ and SPP1^+^ TAMs gene signatures showed different anti- or pro-tumor states.

### Different C1QC^+^ and SPP1^+^ TAMs Gene Signatures Subgroups Showed Variable ICMs Expression and Immunotherapy Response

**Figure 5:**
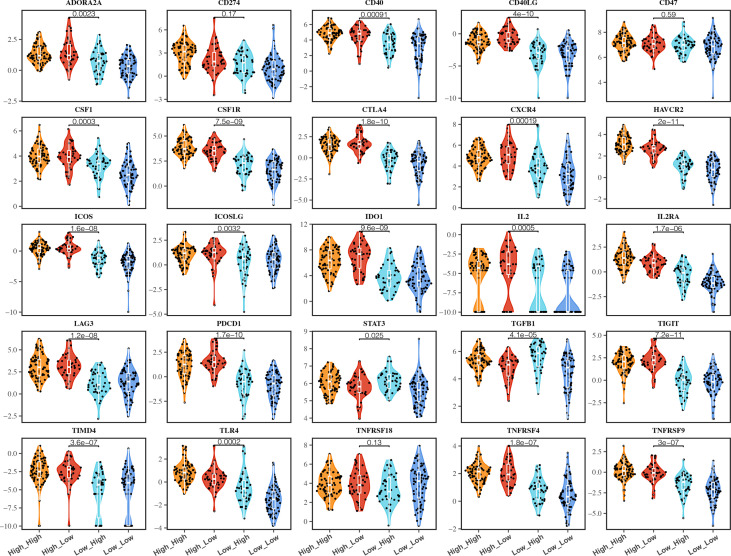
Immune checkpoint molecules (ICMs) expressions in different groups of TCGA cervical cancer patients. Violin plots showing comparison of ICMs expressions among cervical cancer patients grouped by the gene signature expression of C1QC^+^ TAM and SPP1^+^ TAM.

The expressions of ICMs were associated with checkpoint inhibitor immunotherapy response (30, 31). Many ICMs, such as PD1, CTLA4, IDO1, and HAVCR2, were used as the immunotherapy targets in the clinical trials (30). We compared most ICM expression among different C1QC^+^ and SPP1^+^ TAMs gene signatures subgroups. Most (19/25) of the ICMs express higher in patients with C1QC^high^ TAMs gene signatures compared with patients with C1QC^low^ TAMs gene signatures (**Figure 5**). Also, we found that patients with C1QC^high^ and SPP1^low^ TAMs gene signatures had the highest expression of some ICMs (CD40LG, ADORA2A, CTLA4, IL2, LAG3, PDCD1, and TIGIT) compared with the other three subgroups (**Figure 5**), which means these patients may benefit more from ICI immunotherapy. Also, we also notice that the patients with C1QC^high^ and SPP1^low^ TAMs gene signatures showed best OS and DSS (**Figures 2E, F** **)**. We used TIDE (21) to predict response to immunotherapy and found that patients with C1QC^high^ TAMs gene signatures had higher immunotherapy response ratio than those with C1QC^low^ TAMs gene signatures. Patients with C1QC^low^ and SPP1^high^ TAMs gene signatures had the lowest ratio of response to immunotherapy (**Figure S4**). These results suggest that the C1QC^+^ and SPP1^+^ TAMs gene signatures may be used to select cervical cancer patients who will benefit more from ICI immunotherapy.

## Discussion

The development of cervical cancer is reportedly associated with human papillomavirus (HPV) infection, especially HPV intergation (32, 33). On the other hand, immune system defects play a significant role in cancer progress, It is believed that HPV infection triggers a primarily cell-mediated immune response (34, 35). Macrophage percentage was reported to increase linearly with neoplasia progression (36). Some studies showed that higher FIGO stage and lymph node metastasis or lymphangiogenesis usually showed larger counts of M2 macrophages, which were usually associated with poor prognosis (34). However, TAMs are of high heterogeneity, which contain various subsets with different functions. TAMs in different tumors also show different subsets (11, 12). In this study, we evaluated the “traditional” M1/M2 gene signatures and the C1QC^+^ and SPP1^+^ TAMs gene signatures in cervical cancer. We found that C1QC^+^ and SPP1^+^ TAMs gene signatures were more suitable to divide cervical patients into subgroups with distinct clinical outcomes than M1/M2 gene signatures. Our research has three important implications for understanding the role of TAM cells in cervical cancer immunity.

First, we found that C1QC^+^ and SPP1^+^ TAMs gene signatures, but not M1 and M2 gene signatures, could clearly divided TAMs into two subsets in a colon cancer data set and an advanced basal cell carcinoma data set at single cell level. Although we did not have single cell level data to show subsets of TAMs in cervical cancer, we showed that, by using bulk RNA-seq data of cervical cancer from TCGA, C1QC^+^ and SPP1^+^ TAMs gene signatures, but not M1 and M2 gene signatures, could divide cervical cancer patients into subgroups with different prognosis and different tumor stages. Patients with the C1QC^high^ and SPP1^low^ TAMs gene signatures had the lowest ratio of local advanced FIGO stages, whereas patients with the C1QC^low^ and SPP1^high^ TAMs gene signatures had the highest ratio of local advanced FIGO stages. C1QC^+^ and SPP1^+^ TAMs gene signatures were obtained from TAMs; however, they could significantly divide patients into subgroups with distinct clinical outcomes, implying the importance of TAMs in the development of cervical cancer. Further studies are needed to figure out how the TAMs affect cervical cancer development.

Second, cervical cancer subgroups divided by C1QC^+^ and SPP1^+^ TAMs gene signatures showed different immune cell infiltration, with the C1QC^high^ and SPP1^low^ groups have the highest immune cell infiltration, whereas the C1QC^low^ and SPP1^high^ groups had the lowest immune cell infiltration. It was reported that “hot tumors” (with more T cell infiltration) had higher antitumor ability and were more responsive to immunotherapy than “cold tumors” (with none or few T cell infiltration) (37). In our study, we found that patients of the C1QC^high^ and SPP1^low^ group, which had the highest T-cell infiltration, showed the best prognosis, whereas patients of the C1QC^low^ and SPP1^high^ group, which had the lowest T cell infiltration, showed the worst prognosis. Patients with different C1QC^+^ and SPP1^+^ TAMs gene signature patterns showed different T-cell infiltration, implying the effect of TAMs to T cell infiltration. The mechanism behind this phenomenon needs further research.

Finally, we found that many of the immune checkpoint molecules (ICMs) expressed differently in different C1QC^+^ and SPP1^+^ TAMs gene signature subgroups. Generally, patients with C1QC^high^ TAMs gene signatures have higher immunotherapy checkpoint genes expression than those with C1QC^low^ TAMs gene signatures. Since 2015, Clinical trials on different ICIs have been carried out for cervical cancer (38). However, the evidence is still limited to prove the correlation between ICMs and effects of immunotherapy (39, 40). With more clinical research conducted for cervical cancer, our findings may provide valuable information for them.

As mentioned above, our current study is based on TCGA bulk RNA-seq data, which inevitably has some limitations and needs further verification. Therefore, we are now working to verify the gene signatures of C1QC^+^ and SPP1^+^ TAMs in the clinical specimens of patients with cervical cancer at different clinical stages by utilizing single-cell sequencing technology. We believe that the combination of bulk RNA-seq and single-cell sequencing data will help us confirm the gene signatures of C1QC^+^ and SPP1^+^ TAMs in the cervical cancer microenvironment and signaling pathways, which may activate or in-activate in different TAMs subsets. RT-qPCR, FACS, and even IHC could also be used to identify the gene signatures in a large scale of clinical or animal model specimens. It is important to determine the role of C1QC^+^ and SPP1^+^ TAMs subsets in cervical cancer evolution and progression, and some ongoing experiments are in process. It is reported that there are crosstalks between TAMs and T cells, TAMs, and tumor cells. TAMs may interact with CD8^+^ T cells and tumor cells through receptor-ligand pairs, such as SPP1-CD44 (41). The crosstalks between TAMs and CD8^+^ T cells/tumor cells may be validated by using multiplex imaging analysis (41).

In conclusion, C1QC^+^ and SPP1^+^ TAMs gene signatures derived from TAMs can divide cervical patients into subgroups with different prognosis and tumor stage, which may due to different immune cell infiltration. Our findings may help to find suitable treatment strategy for different subgroups of cervical cancer patients.

## Data Availability Statement

The original contributions presented in the study are included in the article/**Supplementary Material**. Further inquiries can be directed to the corresponding author.

## Author Contributions

XL, QZ, GC, and DL designed the study. XL and DL analyzed, interpreted data, and wrote the paper. All authors contributed to the article and approved the submitted version.

## Funding

This work was supported by the National Natural Science Foundation of China (81902653, 81472783, 81472444, 81202061), and Wuhan Municipal health Commission (WX18Q16).

## Conflict of Interest

The authors declare that the research was conducted in the absence of any commercial or financial relationships that could be construed as a potential conflict of interest.

## Supplementary Material

Supplementary Figure 1 Single-cell transcriptome profiling and TAM gene signatures of the Human BCC TME. **(A)** UMAP plot showing major immune cell subsets in human BCC TME. **(B)** UMAP plot of all immune cells colored by enrichment of C1QC^+^ TAM gene signatures. **(C)** UMAP plot of all immune cells colored by enrichment of SPP1^+^ TAM gene signatures. **(D)** UMAP plot of all immune cells colored by enrichment of M1 gene signatures. **(E)** UMAP plot of all immune cells colored by enrichment of M2 gene signatures.

Supplementary Figure 2 M1 and M2 gene signatures in TCGA cervical cancer patients. **(A)** Violin plots showing comparison of M1 gene signatures levels between patients with FIGO stage 1-IB1 and patients with FIGO stage IB2-IVA in TCGA. Two-sided Wilcoxon test. **(B)** Violin plots showing comparison of M2 gene signatures levels between patients with FIGO stage 1-IB1 and patients with FIGO stage IB2-IVA in TCGA. Two-sided Wilcoxon test. **(C)** The Kaplan-Meier overall survival curves of TCGA cervical cancer patients grouped by the gene signature expression of M1 and M2. **(D)** The Kaplan-Meier Disease specific survival curves of TCGA cervical cancer patients grouped by the gene signature expression of M1 and M2.

Supplementary Figure 3 Heatmaps of immune cell infiltration in different groups of TCGA cervical cancer patients. **(A)** Heatmap showing immune cell signatures by XCELL in cervical cancer patients grouped by the gene signature expression of C1QC^+^ TAM and SPP1^+^ TAM. **(B)** Heatmap showing immune cell signatures by CIBERSORT in cervical cancer patients grouped by the gene signature expression of C1QC^+^ TAM and SPP1^+^ TAM.

Supplementary Figure 4 Immunotherapy responses predicted by TIDE in TCGA cervical cancer patients. Immunotherapy responses were predicted by TIDE in cervical cancer patients grouped by the gene signature expression of C1QC^+^ TAM and SPP1^+^ TAM.
