# Supplementary figures and images for: Multi-Omics Analysis Showed the Clinical Value of Gene Signatures of C1QC+ and SPP1+ TAMs in Cervical Cancer
Source: Front Immunol. 2021 Jul 6;12:694801. doi: 10.3389/fimmu.2021.694801 (PMC8290180; doi:10.3389/fimmu.2021.694801)

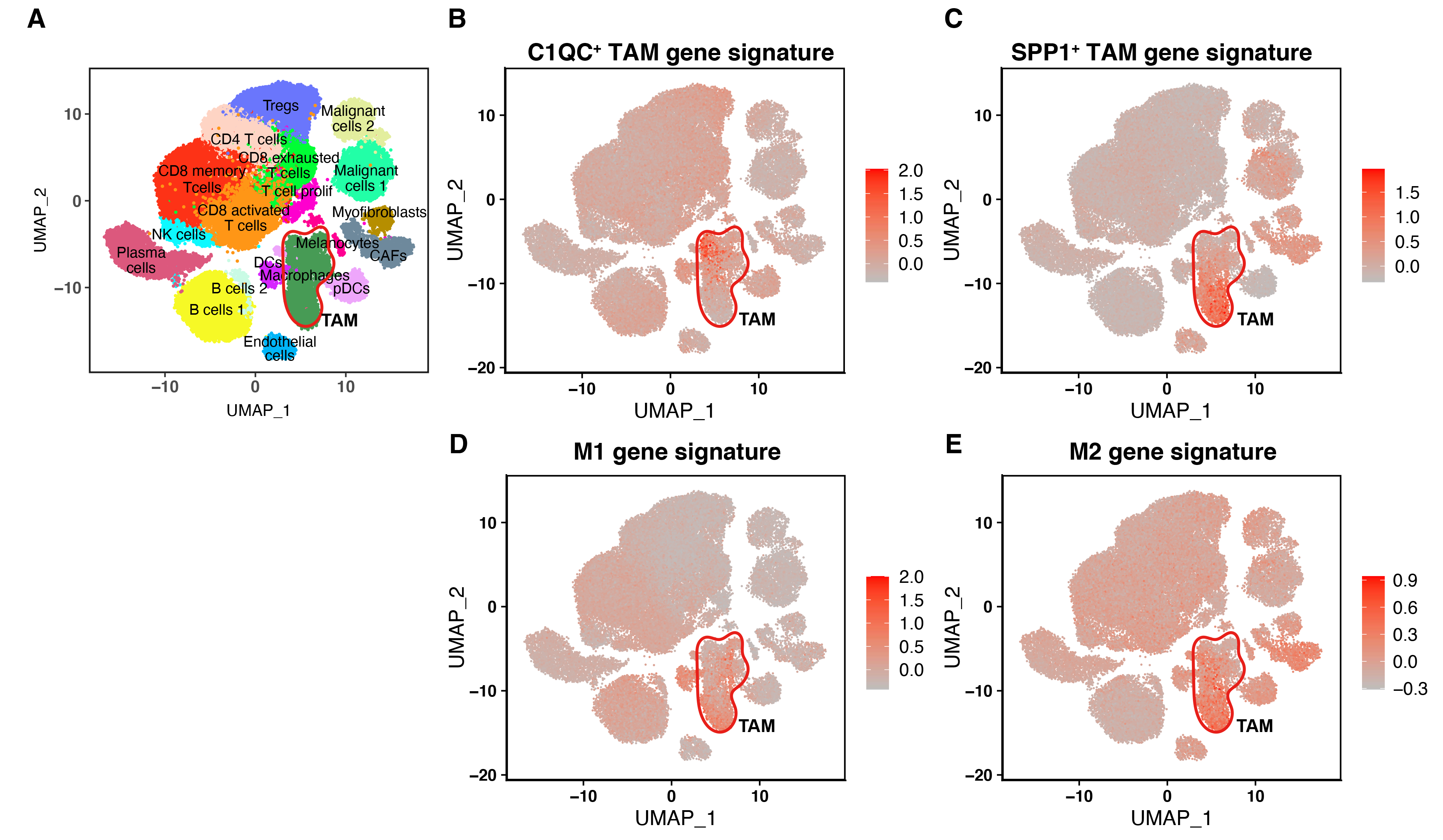

Supplement: Supplementary Figure 1 — Single-cell transcriptome profiling and TAM gene signatures of the Human BCC TME. (A) UMAP plot showing major immune cell subsets in human BCC TME. (B) UMAP plot of all immune cells colored by enrichment of C1QC+ TAM gene signatures. (C) UMAP plot of all immune cells colored by enrichment of SPP1+ TAM gene signatures. (D) UMAP plot of all immune cells colored by enrichment of M1 gene signatures. (E) UMAP plot of all immune cells colored by enrichment of M2 gene signatures. [file Image_1.tif]

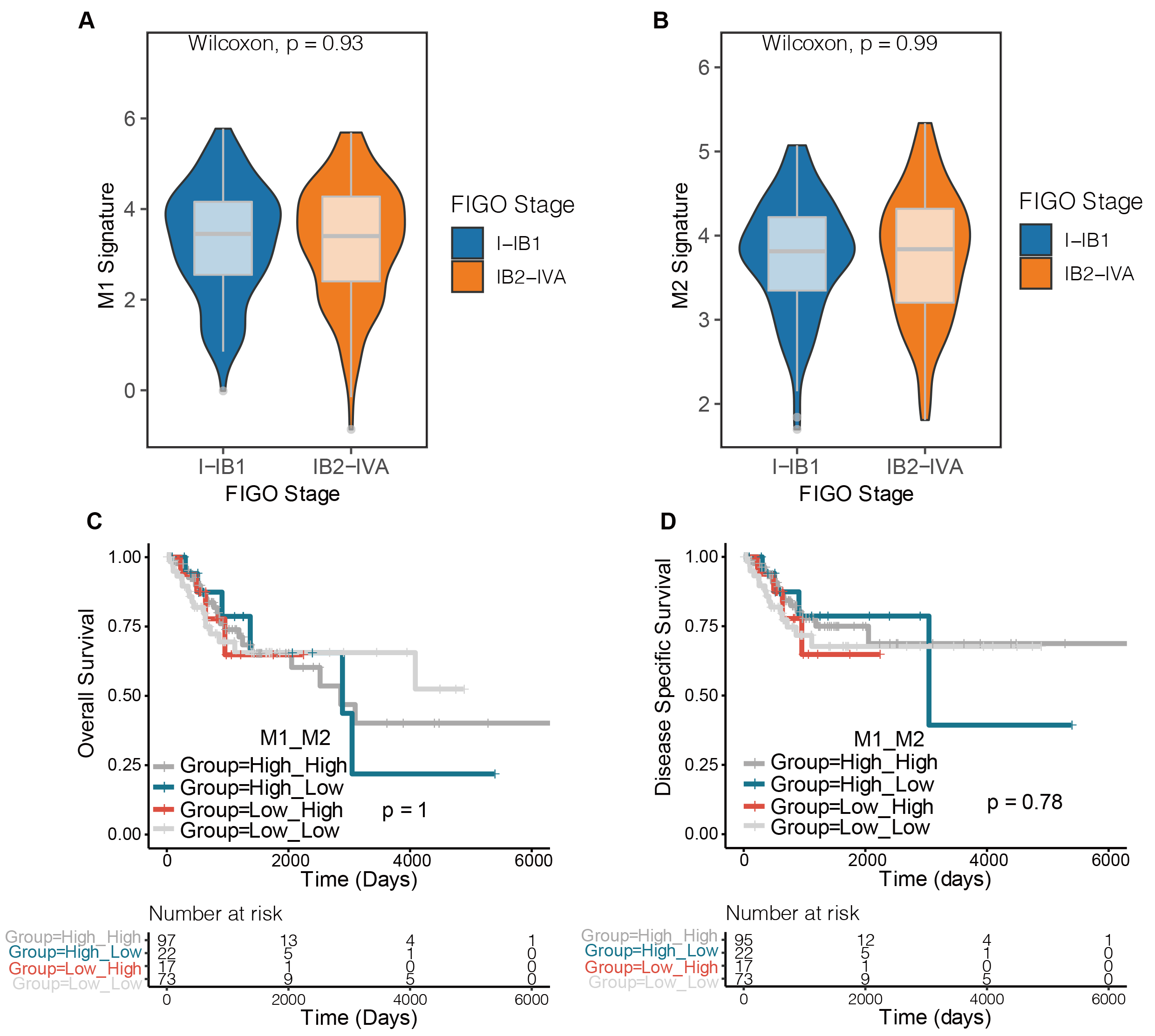

Supplement: Supplementary Figure 2 — M1 and M2 gene signatures in TCGA cervical cancer patients. (A) Violin plots showing comparison of M1 gene signatures levels between patients with FIGO stage 1-IB1 and patients with FIGO stage IB2-IVA in TCGA. Two-sided Wilcoxon test. (B) Violin plots showing comparison of M2 gene signatures levels between patients with FIGO stage 1-IB1 and patients with FIGO stage IB2-IVA in TCGA. Two-sided Wilcoxon test. (C) The Kaplan-Meier overall survival curves of TCGA cervical cancer patients grouped by the gene signature expression of M1 and M2. (D) The Kaplan-Meier Disease specific survival curves of TCGA cervical cancer patients grouped by the gene signature expression of M1 and M2. [file Image_2.tif]

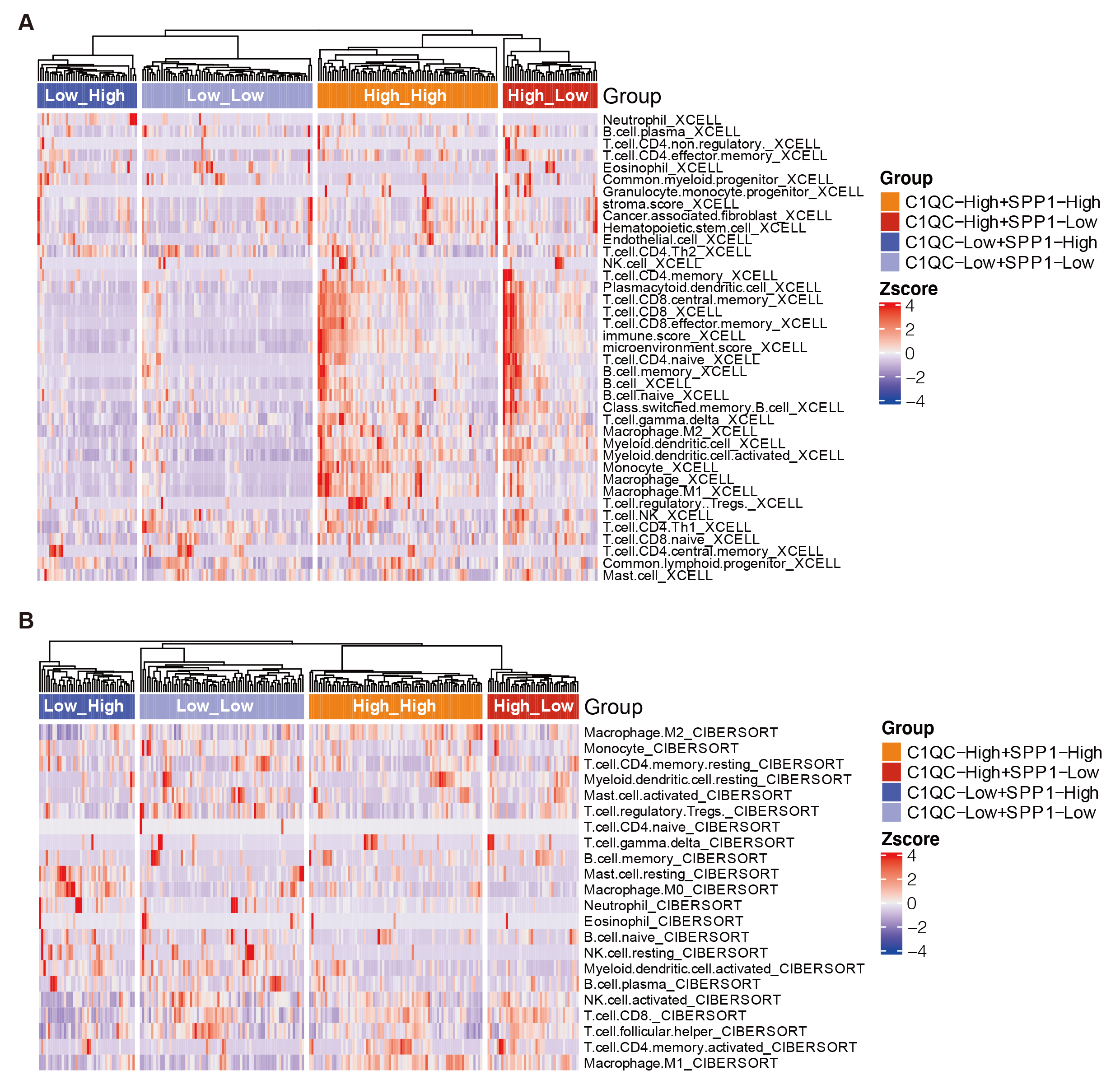

Supplement: Supplementary Figure 3 — Heatmaps of immune cell infiltration in different groups of TCGA cervical cancer patients. (A) Heatmap showing immune cell signatures by XCELL in cervical cancer patients grouped by the gene signature expression of C1QC+ TAM and SPP1+ TAM. (B) Heatmap showing immune cell signatures by CIBERSORT in cervical cancer patients grouped by the gene signature expression of C1QC+ TAM and SPP1+ TAM. [file Image_3.tif]

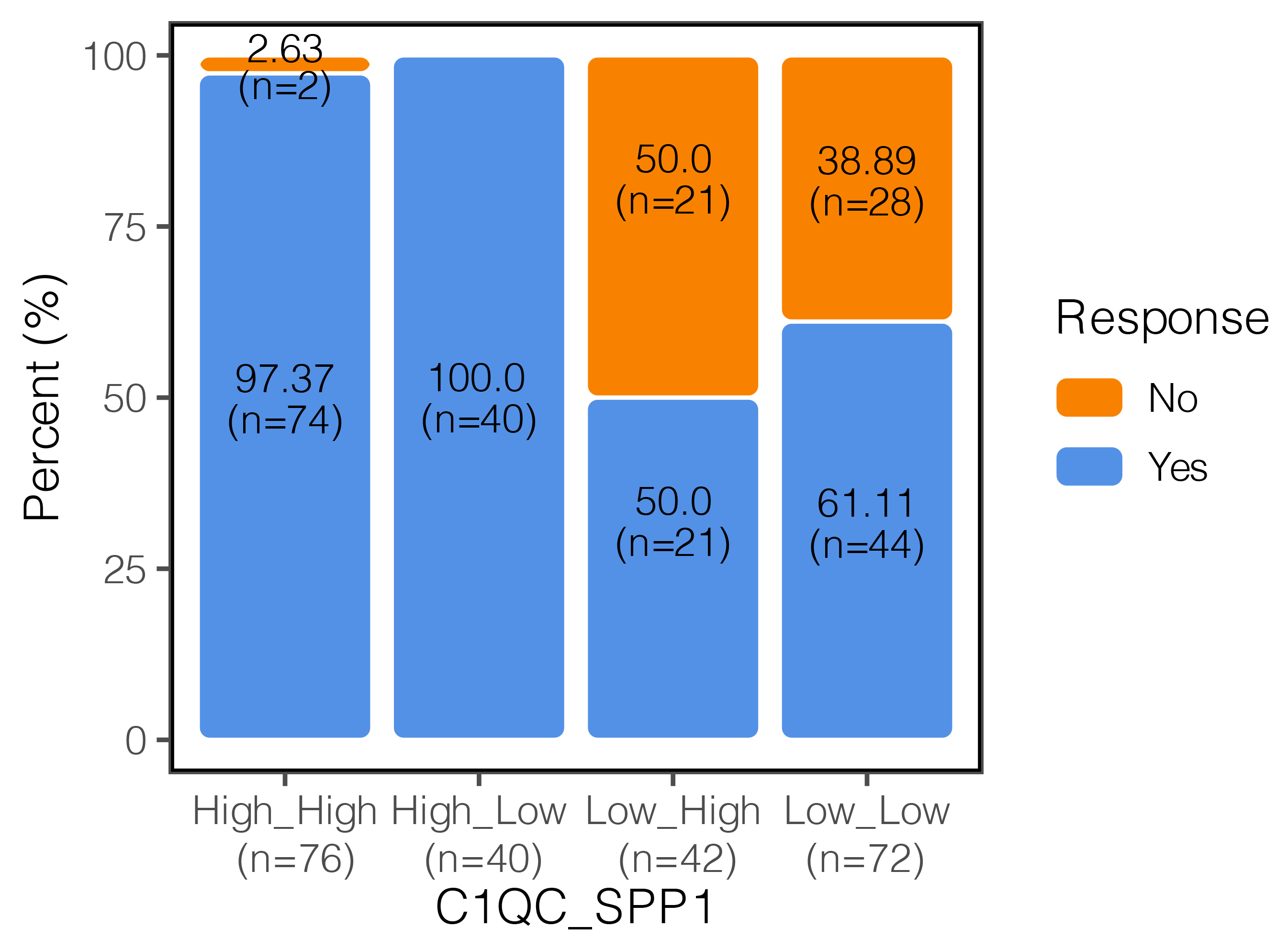

Supplement: Supplementary Figure 4 — Immunotherapy responses predicted by TIDE in TCGA cervical cancer patients. Immunotherapy responses were predicted by TIDE in cervical cancer patients grouped by the gene signature expression of C1QC+ TAM and SPP1+ TAM. [file Image_4.tif]
